# Novel Insights into Autophagy and Prostate Cancer: A Comprehensive Review

**DOI:** 10.3390/ijms23073826

**Published:** 2022-03-30

**Authors:** Davide Loizzo, Savio Domenico Pandolfo, Devin Rogers, Clara Cerrato, Nicola Antonio di Meo, Riccardo Autorino, Vincenzo Mirone, Matteo Ferro, Camillo Porta, Alessandro Stella, Cinzia Bizzoca, Leonardo Vincenti, Marco Spilotros, Monica Rutigliano, Michele Battaglia, Pasquale Ditonno, Giuseppe Lucarelli

**Affiliations:** 1 Department of Emergency and Organ Transplantation–Urology, Andrology and Kidney Transplantation Unit, University of Bari, 70124 Bari, Italy; d.loizzo27@gmail.com (D.L.); nickant.dimeo@gmail.com (N.A.d.M.); dr.marcospilotros@libero.it (M.S.); monica.rutigliano@virgilio.it (M.R.); michele.battaglia@uniba.it (M.B.); pasquale.ditonno@uniba.it (P.D.); 2 Division of Urology, Virginia Commonwealth University Health, Richmond, VA 23298, USA; pandolfosavio@gmail.com (S.D.P.); devin.rogers@vcuhealth.org (D.R.); riccardo.autorino@vcuhealth.org (R.A.); 3 Division of Urology, Università degli Studi di Napoli “Federico II”, 80100 Napoli, Italy; vincenzo.mirone@unina.it; 4 Department of Urology, University of California San Diego, La Jolla, CA 92037, USA; clara.cerrato01@gmail.com; 5 Division of Urology, European Institute of Oncology (IEO), IRCCS, 20141 Milan, Italy; matteo.ferro@ieo.it; 6 Department of Biomedical Sciences and Human Oncology, University of Bari, 70124 Bari, Italy; camillo.porta@uniba.it (C.P.); alessandro.stella@uniba.it (A.S.); 7 Department of General Surgery “Ospedaliera”, Polyclinic Hospital of Bari, 70124 Bari, Italy; cinziabiz84@gmail.com (C.B.); dr.leonardo.vincenti@gmail.com (L.V.)

**Keywords:** prostate cancer, cancer, autophagy, apoptosis, genes, castration-resistant prostate cancer, neoplasia, self-eating

## Abstract

Autophagy is a complex process involved in several cell activities, including tissue growth, differentiation, metabolic modulation, and cancer development. In prostate cancer, autophagy has a pivotal role in the regulation of apoptosis and disease progression. Several molecular pathways are involved, including PI3K/AKT/mTOR. However, depending on the cellular context, autophagy may play either a detrimental or a protective role in prostate cancer. For this purpose, current evidence has investigated how autophagy interacts within these complex interactions. In this article, we discuss novel findings about autophagic machinery in order to better understand the therapeutic response and the chemotherapy resistance of prostate cancer. Autophagic-modulation drugs have been employed in clinical trials to regulate autophagy, aiming to improve the response to chemotherapy or to anti-cancer treatments. Furthermore, the genetic signature of autophagy has been found to have a potential means to stratify prostate cancer aggressiveness. Unfortunately, stronger evidence is needed to better understand this field, and the application of these findings in clinical practice still remains poorly feasible.

## 1. Introduction

Cell homeostasis is a complex set of metabolic processes acting to maintain the cellular environment steady. Among homeostatic processes, autophagy is one of the most important. Also known as “self-eating”, it represents an essential process for the cell, affecting several cellular activities. Autophagy has historically been considered a nonselective process for degradation through the lysosomal system of either older proteins or cytoplasmic components to both recycle building blocks and help to restore the cellular energy balance, especially during nutrient deprivation [1].

Prostate cancer (PCa) is the most frequently diagnosed cancer in Western Europe and occidental countries, recognized as the second leading cause of death after lung cancer [2]. It is well known that autophagy plays a crucial role in cancer development and in response to drugs and xenobiotics [3]. It plays a pivotal role in chemotherapy resistance, and targeting its activity still represents a valid option to improve chemotherapy effectiveness [4]. Recent findings have been conducted on molecular and biological pathways of autophagy. Moreover, new evidence has been found on the autophagic response to medical treatment against prostate cancer (PCa) and how autophagy itself may be affected by anti-cancer drugs.

The aim of this article is to review the most recent findings on autophagy and PCa, focusing on autophagic modulation intended as a potential option to improve PCa treatment effectiveness.

## 2. Autophagic Machinery: Recent Evidence

Autophagy is a high conserved homeostatic process that contributes to the regulation of cellular mass, the proper distribution of organelles, and the removal of cellular toxins and harmful components. The functioning of autophagy is highly complex, and it interacts with several biological processes, such as tissue growth and differentiation, immune system control, and cancer cell withdrawal [5,6]. In response to the energy need of the cells, autophagy is initiated by the combined action of the ATG1 (autophagy-related 1)/ULK1 (Unc-51 such as autophagy activating kinase-1) complex and the Phosphoinositide 3-kinase III (PI3K-III) complex. The nucleation of phagophores is the following step: phagophores phagocytize intracellular cargos generating double-membranous structures called autophagosomes. These autophagosomes eventually fuse with lysosomes to form autolysosomes, wherein the contents are degraded to release amino acids and other metabolic compounds [7].

### 2.1. Autophagic Modulation

Autophagy regulation and autophagosome formation involve several genes and enzymes (Table 1).

A single core machinery site has been discovered in yeast cells, while in mammalian cells, it seems to be controlled in multiple sites in the cytoplasm [8]. One of the main promoters of autophagy is Beclin-1, a protein encoded by the BECN1 gene. This protein takes part in the phosphatidylinositol-3-kinase (PI3K) complex, which mediates vesicle-trafficking processes [9]. Beclin-1 deficiency has been related to solid tumors development. Indeed, its deletion has been associated with a higher risk of malignancies in humans. Furthermore, BECN1 heterozygous mice develop spontaneous cancers [10,11].

**Figure 1:**
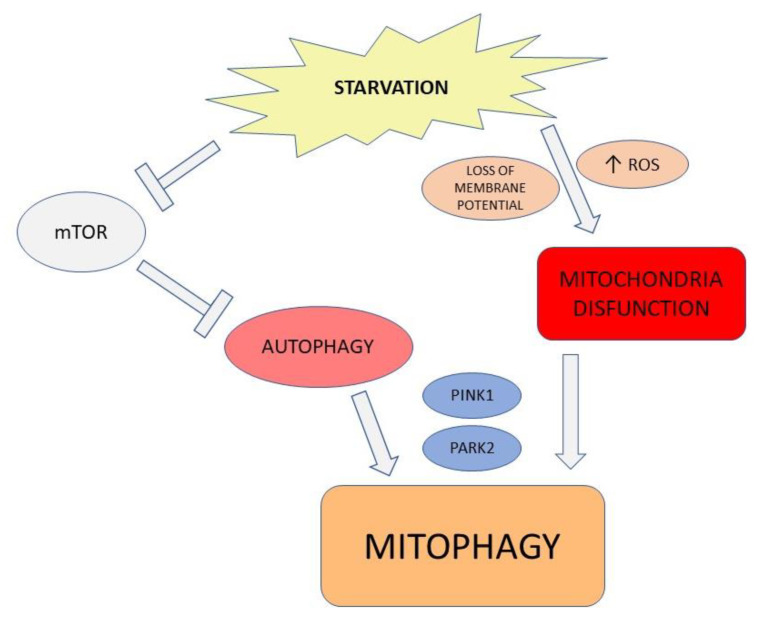
Metabolic activity and mitophagy. Decrease in energetic supply inhibits mTOR activity, which enhances autophagy. The energetic starvation leads to the loss of mitochondria membrane potential, associated to ROS overproduction. This condition activates PTEN-induced putative kinase 1 (PINK1) and the E3 ligase parkin (PARK2), which is able to ubiquitylate mitochondrial outer membrane proteins, providing recognition signals to the active autophagy machinery.

The metabolic activity of the cell counts the mammalian target of rapamycin complex 1 (mTORC1) as a modulator. The energetic plenty switches the metabolic behavior into anabolism, triggered by the mTORC1 complex, which phosphorylates ULK1 and ATG13. The phosphorylated ULK1 complex suppresses autophagic activity [12]. Moreover, autophagy controls mitochondria activity under stress conditions (Figure 1) as well. Dysfunctional mitochondria lose membrane potential, leading to the activation of PTEN-induced putative kinase 1 (PINK1). PINK1 activates the E3 ligase parkin (PARK2) to ubiquitylate mitochondrial outer membrane proteins, providing recognition signals to the autophagy machinery in order to dismantle them. The selective elimination of damaged mitochondria is a strategy to maintain a high mitochondrial quality associated with a decrease in metabolic stress and ROS production [13,14].

Calcium/calmodulin-dependent protein kinase kinase 2 (CaMKK2) belongs to the serine/threonine-specific protein kinase family. CaMMKs are pivotal components of multiple metabolic pathways which regulate glucose metabolism, adipogenesis, nutrient intake, and inflammation. Furthermore, its activity has been related to cancerogenesis. Therefore, CaMKK2 results overexpressed in prostate cancer as well, in part due to androgen-receptor hyperactivity [15]. Recently, it has been demonstrated that knocking down CAMKK2 leads to perturbations indicative of vesicle trafficking disruption. These findings are consistent with the role of autophagy in cancer development, as CAMKK2 provides sustain to cell proliferation through effects on organelle integrity and membrane trafficking [16].

However, the relationship between autophagy and cancer development is complex, and some contradictions may concern. It has been shown that the disruption of Beclin-1 could increase autophagic activity, improving longevity and lifespan in mice [17]. This may be possible considering the inhibitory activity which Beclin-1 exerts on B-cell lymphoma 2 (Bcl2), promoting cellular survival. These findings are consistent, however, with the increased probability of cells developing malignancies when the control of apoptosis is not accurate.

### 2.2. Autophagy and Apoptosis

Notably, Beclin 1 is a BH3-only protein that is directly antagonized by the binding of Bcl-2/Bcl-xL. It has been shown that the endoplasmic reticulum pool of Bcl-2/Bcl-xL is responsible for the regulation of autophagy. This may suggest that the anti-autophagic and antiapoptotic effects of Bcl-2/Bcl-xL are regulated within the cell depending on their localization [18]. This evidence shows how autophagy is multi-faceted and closely connected to apoptosis, also considering the control exerted by apoptosis on autophagy or on their molecular intermediates, which both processes share as modulators [19]. Moreover, under certain cellular conditions, autophagy can promote cell death by either serving as an alternative cell death mechanism or enabling the induction of apoptosis. For example, mouse embryonic fibroblasts (MEFs) from Bax/Bak double-knockout mice fail to undergo apoptosis in response to the DNA-damaging agent etoposide. On the other hand, they are observed to induce massive autophagy followed by Atg gene-dependent cell death [20].

**Figure 2:**
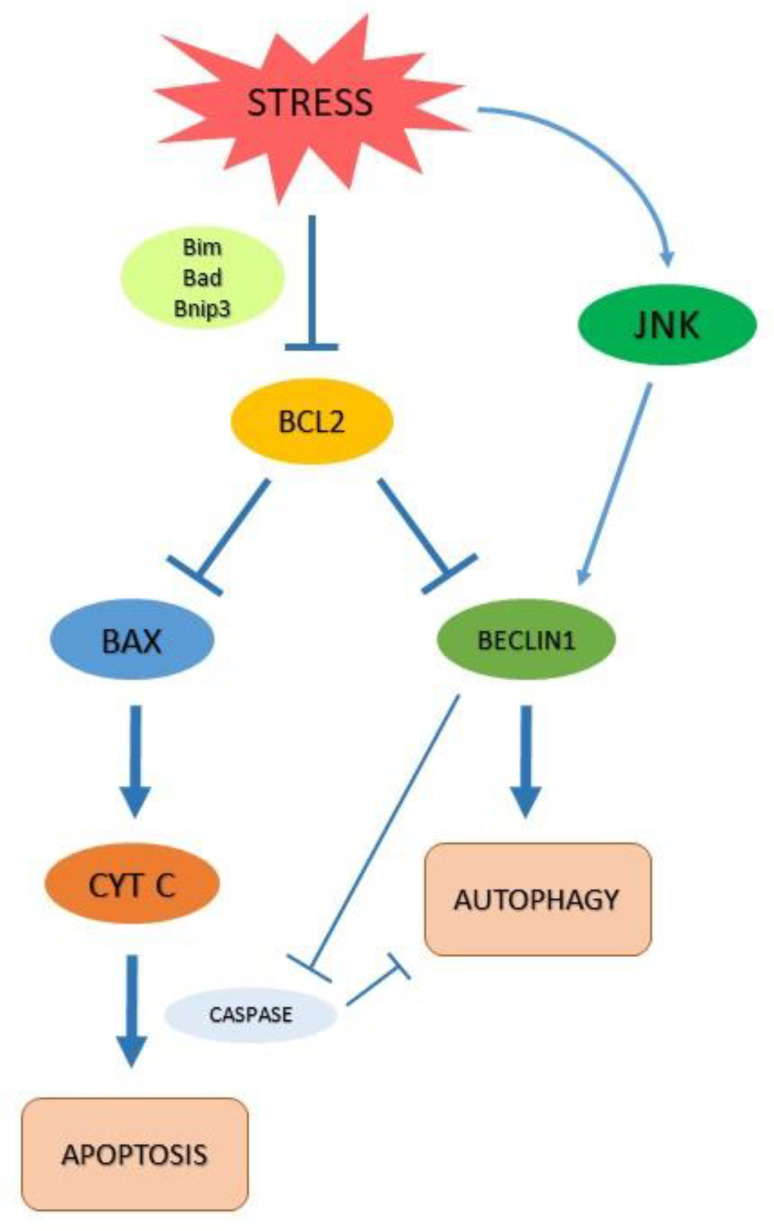
Autophagy and apoptosis interplay. Stress conditions can inhibit Bcl2 family through the phosphorylation of Bim/Bad/Bnip3. This condition results in liberation of Beclin1 and induction of autophagy, enhanced by the activation of c-Jun N-terminal kinases (JNKs). Subsequently, the oligomerization of the pro-apoptotic proteins BCL-2 antagonist killer 1 (BAK) and BCL-2-associated X protein (BAX) results in mitochondrial outer membrane permeabilization (MOMP), which leads to the release of cytochrome C (Cyt C). Cyt C will form a complex with pro-caspases, resulting in Caspases activation and Cell death. Apoptosis and autophagy may also inhibit each other through protein cleavage.

Furthermore, the Bcl-2 protein family provides a molecular link between the intrinsic pathway of apoptosis and autophagy (Figure 2). Another example of this complex interaction is given by Bildik and colleagues [21]. These authors have recently reviewed the role of the RAS GTPase 3 known as DIRAS3 (Distinct Subgroup of The Ras Family Member 3), in several cancers, including PCa. DIRAS3 is a unique endogenous RAS inhibitor that binds directly to RAS, disrupting RAS dimers and preventing RAS-induced transformation. DIRAS3 is an essential key in autophagy and triggers this process through multiple mechanisms: it acts (i) blocking PI3K/AKT signaling to downregulate mTOR and therefore decreasing ULK1 phosphorylation to induce autophagy; (ii) displacing Bcl-2 from Beclin1 to form the autophagy initiation complex; (iii) inhibiting RAS/MAPK (mitogen-activated protein kinase) and PI3K, maintaining FOXO3a (Forkhead box O3a) in the nucleus to induce critical gene expressions that participate in autophagy such as microtubule-associated protein light chain 3 (LC3) and Atg4. In this scenario, it has been shown that DIRAS3 induction of autophagy facilitates the survival of dormant cells in a nutrient poor-environment [21]. This is consistent with evidence that considers autophagy as a self-protective mechanism acted by cancers cells to balance their metabolic rate and maintain their replicative power. Moreover, autophagy is central in cancer biological processes, providing an energetic supply and metabolic compounds to escape regulators of apoptosis. Cancer cells have a high-rate metabolism, seldom working in hypoxic conditions or metabolic starvation. Conversely, autophagy is essential to provide metabolic supply even in low nutrient or oxygen levels [22]. Upregulation of autophagy in cancer tissue is considered protective for this purpose. Autophagic response has been investigated in PCain relation to androgen deprivation therapy (ADT) and to chemotherapy (in particular to taxanes action).

## 3. Autophagy and Prostate Cancer

PCa is often diagnosed de novo among men in advanced or metastatic stages. The standard of care in advanced disease is androgen deprivation therapy (ADT), considering the crucial role of the androgen receptor (AR) in PCa cell activity [23]. Although ADT is initially effective in slowing down the disease progression, it could fail within 2–3 years when the disease progresses to a stage referred to as castration-resistant prostate cancer (CRPC). Interestingly, despite the ineffectiveness of ADT in CRPC, the majority of prostate cancers are still driven by AR as a result of several AR reactivation mechanisms [24,25,26,27,28,29,30,31,32,33,34,35,36].

**Figure 3:**
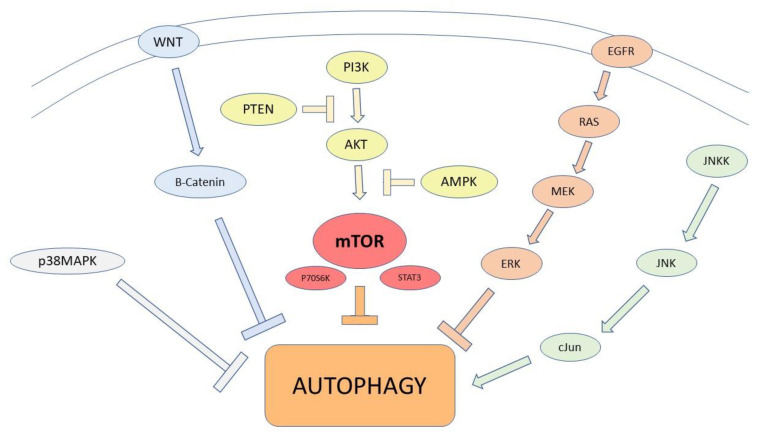
Main pathways involved in autophagy regulation. PTEN and AMPK downregulate the PI3K/AKT/mTOR signaling pathway, to enhance autophagy. mTOR suppresses autophagy acting via P70S6K and STAT 3 expression. The activation of the EGFR/Ras/MEK/ERK pathway, JNK/cJun, and p38MAPK signaling pathways promotes autophagy. The Wnt/β-catenin signaling pathway attenuates Beclin1-mediated autophagy.

Cancer cell autophagy may involve many pathways, and some of the transduction pathways involved are herein reported (Figure 3) [37].

As aforementioned, mTORC is crucial in autophagy regulation and cancer development. Decreased autophagy as a result of a disrupted activation of mTORC1 may participate in carcinogenesis. In fact, liver-specific phosphatase and Tensin Homolog (PTEN) or Tuberous Sclerosis Complex 1 (TSC1) knockout mice exhibit decreased autophagy caused by a constitutively activated mTORC1, leading to the development of hepatocellular carcinoma [38]. Although autophagy acts as a tumor suppressor in non-tumor cells and during the early stages of tumor cell development, its activation becomes crucial for cancer cells to survive once tumor mass is established. Cancer cells are characterized by an increased metabolic demand, intended as a higher energetic rate and building block utilization. They enhance the autophagic flux in order to bear the metabolic starvation, becoming dependent on the energetic supply [39].

**Figure 4:**
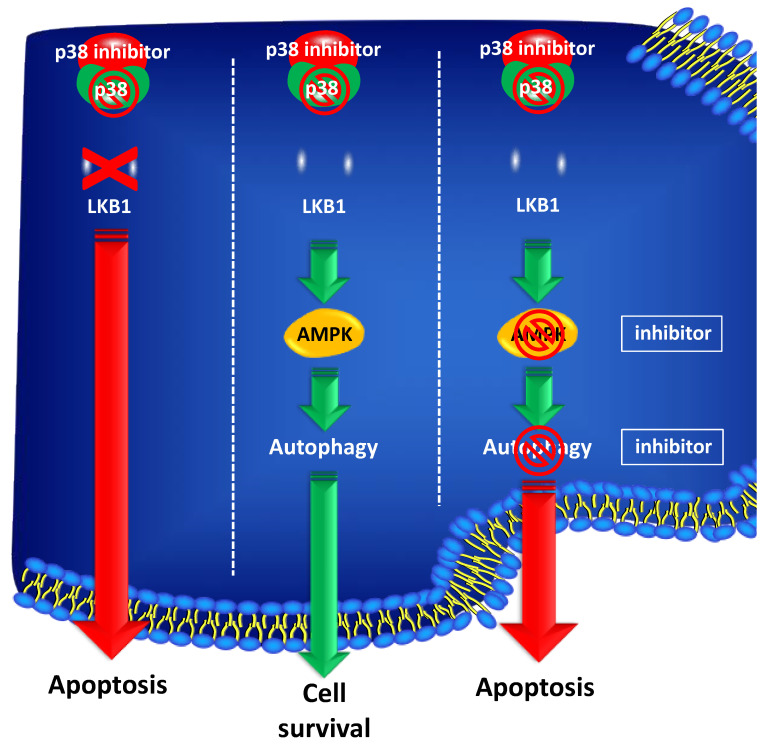
STK11/LKB1 expression is a predictive marker of response to p38MAPK inhibition in PCa. PCa cells with low/absent STK11/LKB1 expression undergo cell death after treatment with p38MAPK inhibitors. PCa cells expressing high levels of STK11/LKB1, show resistance to p38MAPK blockade, by activating autophagy. Conversely, inhibition of autophagy or of AMPK, triggers apoptosis in STK11/LKB1 expressing cells treated with p38MAPK inhibitors.

Other genes related to autophagy involved in PCa carcinogenesis have been identified upstream of mTORC activity. *STK11*, for example, encodes for a tumor suppressor serine-threonine kinase, also known as LKB1, which regulates several cell functions, including proliferation, cell cycle arrest, differentiation, energy metabolism, and cell polarity. The main effector of STK11/LKB1 is AMPK (adenine monophosphate-activated protein kinase), which is a central metabolic modulator in normal and in neoplastic cells. AMPK activity cross talks with the phosphoinositide 3-kinase (PI3K), MTOR, and mitogen-activated protein kinase (MAPK) pathways [40]. It has been shown that STK11/LKB1 expression is lost throughout PCa progression from normal to neoplastic tissue. Histologically, prostatic carcinogenesis is characterized by a progressive transition from normal epithelium to high-grade PIN (Prostatic intraepithelial neoplasia) to invasive PCa. STK11/LKB1 expression is evident only in normal or atrophic cells, while dysplastic cells show no staining by immunohistochemistry [41]. In addition, it was shown that inhibition of p38MAPK affected PCa cells’ survival depending on the expression of STK11/LKB1. In particular, PCa patients with no STK11/LKB1 expression could benefit from a therapeutic approach based on p38MAPK inhibitors in addition to ADT. Conversely, PCa patients with high expression of STK11/LKB1 should be candidates for dual kinase inhibition targeting both p38MAPK and AMPK or for a treatment targeting the autophagic machinery (Figure 4).

These findings are consistent with the evidence, stating that the balance between the apoptotic and autophagic machinery in PCa and other solid malignancies is regulated by the crosstalk between STK11/LKB1 and MAPK [42].

In PCa, progression is mediated by effects of androgen receptor (AR) partially related to autophagic modulation. AR exerts its action on several targets, involving cell replication, energy employment, and subsequently autophagy. Therefore, it has been shown that CaMKK2 is a target gene of AR in prostate cancer. Consistent with the pro-survival role of CaMKK2-mediated autophagy, it has been observed that CaMKK2 knockdown tumors displayed increased necrosis, especially in areas less provided with energetic sustenance. These data may suggest that CAMKK2 is required for PCa progression in vivo by enabling cells to withstand nutrient-deficient tumor microenvironment [43].

Moreover, PCa also shows deep molecular interaction between autophagy and apoptosis. Tumor necrosis factor-related apoptosis-inducing ligand (TRAIL) is a member of the tumor necrosis factor (TNF) family. It is known that TRAIL is able to trigger cancer cells’ death by binding the death receptors (DR-4 and DR-5) and by recruiting FADD (Fas Associated Via Death Domain) and caspase-8 to create the death-inducing complex [44]. It has been demonstrated that TRAIL acts synergistically to trigger autophagy in PCa cells, as revealed by autophagosome formation and LC3B-II accumulation [45].

Recently, the impact of an autophagic genetic signature on the susceptibility and prognosis of PCa has been investigated. Cheng et al. have identified differentially expressed autophagy-related genes (DEARGs) based on The Cancer Genome Atlas (TCGA) Prostate Adenocarcinoma (PRAD) dataset. They identified sixteen DEARGs, and seven of them were associated with PCa overall survival (OS). Moreover, clinical characteristics were associated with three prognostic genes (*NPC1, BNIP3, TP53*): the elevated expression of NPC1 (Niemann–Pick C1 protein) and BNIP3 (BCL2 Interacting Protein 3) was dramatically associated with advanced pathological T stages; in addition, overexpression of NPC1 was significantly related to higher ISUP grades [46]. Similarly, another study conducted by Hu and colleagues has evidenced more than twenty ARGs affecting disease-free survival (DFS) related to T status, N status, and Gleason score [47]. Data were obtained by The Human Autophagy Database: they identified more than 230 ARGs and compared them to differentially expressed ARGs extracted from The Cancer Genome Atlas (TCGA) database. Data are consistent with previous evidence conducted on biopsy specimens. In fact, it has been investigated the relation between cancer aggressiveness and the expression of the four major autophagy proteins (the microtubule-associated protein 1 light chain 3A–LC3A, LC3B, Beclin 1, p62, lactate dehydrogenase 5–LDH5). The extensive cytoplasmic expression of LC3A, LC3B, and p62 (present in >50% tumor cells per section) was significantly associated with LDH5 levels and a high Gleason score. Furthermore, extensive Beclin-1 overexpression was significantly linked with extra prostatic invasion [48]: these findings may be promising to employ ARGs and phenotypical expression of autophagy as potential prognostic biomarkers.

## 4. Prostate Cancer Treatment and Autophagy

The majority of cancer treatments, as well as most new drugs, may affect autophagy (Table 2), usually increasing the autophagic flux. Autophagy has been developed as a protective system for cells under stressors. Therefore, autophagy may seem a natural response if we consider chemotherapy as a common stress agent. However, tumors are often subjected to nutritional stress, hypoxia, and other stimuli such as physical strain and lack of survival signals from the microenvironment. All these stimuli regulate autophagy; indeed, we should consider the response to treatments as a result of these variables taken together [49,50].

Studies have demonstrated that persistent AR signaling is the key driver in the progression to CRPC. In this scenario, cells acquire the ability to activate AR signaling either through AR gene amplification, AR mutation, constitutively active AR splice variants, or increased intratumor androgen production [15]. Autophagy can be used by cancer cells to prolong their survival under harsh conditions of metabolic stress induced by various treatment modalities, such as castration therapy. Moreover, evidence suggests that autophagy itself may promote castration resistance. It has been demonstrated that the expression of autophagy regulation molecules (CAMKK2, AMPK, and ULK1) is related to the prognosis and progression of men with prostate cancer. Furthermore, inhibitors of AMPK-ULK1 signaling blocked autophagy, cell growth, and colony formation in LNCaP cells. Taken together, these findings suggest that the CAMKK2-AMPK-ULK1 signaling cascade may promote PCa and castration resistance by increasing autophagy [51,52]. To the best of our knowledge, in vitro studies have been conducted to counteract autophagy in the castration resistance process. However, their clinical employment lacks feasibility.

Abiraterone and enzalutamide are currently employed in castration-resistant PCa, acting with different mechanisms of action. Enzalutamide (MDV-3100, ENZA) is a molecule that exerts multiple effects on androgen signaling, including: (i) blockage of testosterone binding to AR, (ii) prevention of AR nuclear translocation, and (iii) blockage of AR binding to DNA. It has been proven that ENZA administration triggers an autophagy cascade in the C4-2B subline of LNCaP cells, mediated by the AMPK activation and the mTOR suppression. This subset of cells is able to grow in castrated hosts, reproducing the behavior of CRPCa in humans. Furthermore, it has been shown that targeting AMPK in cells exposed to ENZA could significantly inhibit autophagy and could promote cell death. These findings suggest that autophagy is an important survival mechanism in CRPCa [53].

However, their effects on cells go towards the simple action on androgen receptor activity. It has been proven that both abiraterone and enzalutamide can inhibit PCa proliferation and promote apoptosis through mitophagy regulation. The importance of mitophagy in drugs action is strong: it has been evidenced that the addition of mitophagy inhibitor Mdivi-1 (mitochondrial division inhibitor 1) could conversely elevate proliferation and constrain apoptosis of PCa cells [54]. However, the inhibition of autophagy has been shown to increase the effectiveness of abiraterone. Mortezavi and colleagues have demonstrated that levels of ATG5 and LC3II were increased in PCa cells treated with abiraterone, highlighting the upregulation of autophagy. They have also shown that the addition of autophagy inhibitors could significantly impair cell viability, increasing apoptosis [55]. Same evidence has been given studying apalutamide, another androgen-receptor antagonist employed in castration-resistant PCa. It has been demonstrated that the apalutamide treatment of LNCaP cells leads to autophagy, which exerts a high anti-tumor effect if combined with autophagy inhibitors, such as 3-methyladenine or siRNAs [56]. The LNCaP cell line (Lymph Node Carcinoma of the Prostate) derives from human prostate adenocarcinoma cells from a lymph node metastasis. Highly sensitive androgen receptors are expressed in the cytosol of LNCaP cells, making LNCaP a highly androgen-sensitive cell line. The androgen receptor in the LNCaP cells contains a single point mutation changing the sense of codon 868 (Thr to Ala) in the ligand-binding domain [57]. Nevertheless, the androgen receptor alone cannot completely explain autophagy regulation. For example, PCa cells lines lacking androgen receptors can also increase autophagic flux under ADT. This may be explained by the disruption in further proto-oncogenes such as PTEN or by the mutation of tumor suppressors p53, associated with the aberrant expression of downstream androgen receptor targets [58].

Docetaxel is a microtubule-stabilizing agent, and it is currently a standard of care in men with metastatic PCa or in CRPCa. Several studies have shown a variable degree of autophagic activity during taxane-based therapies. Some studies have demonstrated that taxanes therapy may induce autophagy, whilst others have shown that autophagy is reduced under taxanes therapy [58,59]. However, in breast cancer cells, it is well proven that the autophagic response to taxanes is context-dependent, depending on the duration of the treatment and on environmental factors which contribute to the degree of autophagic activity [59]. Data seem to be controversial, but it strengthens the concept of the versatility of autophagy.

In this scenario, it is well known that autophagy plays a pivotal role in taxanes resistance. Recently, it has been confirmed that docetaxel-resistant PCa cells enhance autophagic flux via upregulation of Forkhead box protein M1 (FOXM1) expression. Moreover, the knockdown of FOXM1 sensitizes the cells to docetaxel both in vitro and in vivo, associated with a decreased autophagic flux and a lower number of autophagosomes [60].

Prostate-specific membrane antigen (PSMA)–targeted radioligand therapy (RLT) with 177Lu or 225Ac yields clinical responses in 57–76% of PCa patients [61,62]. Poor understanding of the underlying resistance mechanisms represents a key barrier to the development of more effective RLT. In this scenario, autophagy may play a relevant role. Recently, it has been demonstrated that the activation of the ATG5-mediated autophagy in response to a lack of glutamine is a tumor survival strategy to withstand radiation-mediated cell damage. In combination with autophagy inhibition, the blockade of glutamine metabolism might be a promising strategy for PCa radiosensitization [63]. Furthermore, the activation of the PI3k/mTOR pathway has been linked to the development of radioresistance, and multiple studies have investigated mTOR inhibitors for their potential radiosensitizing effects. However, the use of mTOR inhibitors as radiosensitizers for RLT remains controversial, as preclinical studies investigating their efficacy have reported contrasting results [64].

## 5. Autophagic Modulation in PCa Treatment

Several studies have been conducted to counteract autophagy in an attempt to restore the sensitivity to docetaxel or ADT. The results are promising in preclinical models, considering that the inhibition of autophagy enhances cell death under anti-cancer treatment. Current research has been shifted into clinical trials to confirm the results of preclinical reports in patients with refractory malignancies [58]. In vitro, inhibitors of autophagy may also include 3 methyladenine (3-MA) and siRNAs. However, siRNAs have been relegated to preclinical studies as confirmatory tools, and they are not currently employed in clinical practice. Alternatively, more clinically applicable inhibitors of autophagy may include chloroquine and its derivative hydroxychloroquine [65]. An ongoing clinical trial may confirm the feasibility and the efficacy of chloroquine employment (NCT04011410, NCT00726596) to improve clinical outcomes. Recently, it has also been investigated by Erkisa et al. the efficacy of chloroquine combined with metal-based chemotherapeutic drugs, such as palladium [66]. They have shown that the palladium complex increases apoptosis in PCa cells. Indeed, pre-treatment with chloroquine potentiated apoptosis via a mitochondria-mediated pathway, and it decreased PI3K/AKT/mTOR-related protein expressions.

Other inhibitors have been investigated in the research field, including metabolic modulator as metformin or fenofibrate. Metformin is an oral biguanide, and it is currently employed for diabetes mellitus II. It is well known for its activity as an autophagy inhibitor as its mechanism of action includes the inhibition of mTOR via AMPK activation. This trigger should enhance the autophagic activity; however, its effect inhibits autophagy blocking Beclin-1 despite AMPK activation [67]. However, metformin exerts an environment-dependent inhibition of autophagy activity. For this purpose, a recent work conducted by Chen et al. demonstrated that metformin might enhance autophagy in PCa cells. Indeed, they have shown that metformin inhibits cell proliferation through the activation of the AMPK pathway, which is associated with a higher autophagic flux and an increased apoptotic activity [68]. These results are consistent with the multi-faceted effects of autophagy to determine cell viability.

Fenofibrate has also been employed as a modulator of autophagy in docetaxel-resistant PCa cells. In fact, a recent study has shown that the association of fenofibrate with docetaxel induces autophagy in PCa cells and make them sensitive to taxanes [69]. Fibrates interfere with the metabolic balance of the cell, leading to an energy-depleted environment. These findings suggest that autophagic induction is an attempt to restore the energetic balance or to quicken cell death [69].

Nevertheless, the clinical response of these drugs and their clinical employment are still poorly feasible. For example, pantoprazole was one of the drugs previously identified as a potential chemotherapy adjuvant. Indeed, it was demonstrated that high-dosed pantoprazole was effective in inhibiting autophagy and preventing taxanes resistance in several solid cancers [70]. A phase II trial (PANDORA) was conducted for this purpose to prove the effectiveness of pantoprazole to enhance the efficacy of docetaxel in metastatic CRPCa. Unfortunately, the resultant clinical activity was not sufficient to meet the predefined target to warrant further testing, even if the treatment was well tolerated in the cohort [71].

## 6. Conclusions

Insights on PCa biology are continuously evolving. Data on autophagy functioning are complex and may seem conflicting, especially if we consider the deep interaction between autophagy and apoptosis. Herein we discussed novel findings on autophagy machinery to better handle PCa therapeutic response and PCa chemotherapy resistance. Depending on the cellular context, autophagy could play either a detrimental or a protective role in PCa survival due to its complex modulation and pleiotropism. Evidence is promising on autophagy modulation in vitro and its employment as a biomarker. This context may find new strategies in PCa therapy or in prognostic stratification. However, evidence still lacks clinical feasibility and effectiveness. The hope is that ongoing trials could elucidate the role of autophagy modulators to improve therapeutic strategies. The genetic signature of autophagy could also be employed in clinical stratification and in prognostic assessment. Further investigation should be conducted in this field to provide potentially tailored treatment and to create diagnostic paradigms based on autophagic activity.

## Figures and Tables

**Table 1:** Main genes involved in autophagosome regulation machinery.

Functional Step	Gene	Function
Induction of autophagy	mTOR	Negative regulator of autophagy
	ULK1	Part of the complex ULK1 kinase
	ATG13	
	RB1CC1	
Autophagosome formation	ATG 2	Components of ATG9-WIPI complex and Vps34-beclin1 class III PI3-kinase complex
	ATG 9	
	WIPI1/2	
	PI3KC3	
	PI3KR4	
	BECN1	
	ATG14	
	ATG12	
	ATG5	
	ATG7	
Autophagosome maturation	ATG16	LC3/ATG8 conjugation
	ATG10	
	CAMKK2	
	MAP1LC3B	
	ATG8	
Autophagosome/lysosome fusion and degradation	ATG3	Ubiquitin-like conjugation systems
	ATG4	
	TFEB	Regulation of lysosomal genes and fusion with autophagosome
	RAB7	

**Table 2:** Summary of PCa drugs interfering with autophagic activity.

Drug	Mechanism of Action	Effect on Autophagy
Abiraterone	Blocking testosterone biosynthesis (CYP17)	Triggering autophagy and mitophagy
Enzalutamide	Blocking testosterone binding to AR	Triggering autophagy via mTOR inhibition
	Preventing AR nuclear translocation	
Apalutamide	Blocking testosterone binding to AR	Triggering autophagy
	Preventing AR nuclear translocation	
Docetaxel	Inhibition of microtubular depolymerization	Triggering autophagy via upregulation FOXM1 expression
	Phosphorylation and inactivation of the bcl-2 protein	
